# Clinical characteristics and short-term outcomes of Japanese encephalitis in pediatric and adult patients: a retrospective study in Northern China

**DOI:** 10.3389/fneur.2023.1135001

**Published:** 2023-04-20

**Authors:** Fangyuan Zhang, Guangyin Xu, Xiaoyu Zhang, Yue Li, Dong Li, Chunjuan Wang, Shougang Guo

**Affiliations:** ^1^Department of Neurology, Shandong Provincial Hospital, Shandong University, Jinan, Shandong, China; ^2^Department of Neurology, Linyi People's Hospital, Linyi, Shandong, China; ^3^Department of Neurology, Shandong Provincial Hospital Affiliated to Shandong First Medical University, Jinan, Shandong, China

**Keywords:** Japanese encephalitis, adult, children, complications, short-term outcome

## Abstract

**Objective:**

The study aimed to compare the clinical characteristics and short-term outcomes of pediatric and adult Japanese encephalitis (JE) patients in order to find out the differences.

**Methods:**

From August 2006 to October 2019, 107 patients (62 pediatric patients and 45 adult patients) with JE were enrolled. Clinical characteristics and short-term outcomes were analyzed. The short-term outcome of each patient was defined as a good outcome or poor outcome according to their Glasgow Coma Scale (GCS) scores (GCS > 8 vs. GCS ≤ 8) at discharge.

**Results:**

As for acute complications, the incidence of pulmonary infection was higher in 25 adults (25/45, 55.6%) than in 19 children (19/62, 30.6%; *P* = 0.01). Upper gastrointestinal bleeding was more common in patients with pulmonary infection, with 10 of these patients experiencing the symptom (10/44, 22.7%) compared to only one patient without pulmonary infection (1/63, 1.6%; *P* = 0.001). The proportion of mechanical ventilation and admission to the intensive care unit (ICU) for supportive care was higher in patients with pulmonary infection than in patients without infection (*P* < 0.001, *P* = 0.008, respectively). The GCS scores at discharge in patients with pulmonary infection (7, 4–12.75) were lower than in patients without pulmonary infection (14, 10-14; *P* < 0.001). Although the GCS scores at the admission of children (9.5, 7–13) were similar to that of adults (7, 6–13), the GCS scores at the discharge of adults (7, 3.5–13) were lower than that of children (13, 10.75–14; *P* < 0.001).

**Conclusion:**

The short-term outcome of JE was worse in adults. Pulmonary infection was correlated with a high incidence of upper gastrointestinal bleeding, mechanical ventilation, and ICU hospitalization in JE. Pulmonary infection is a prognostic predictor of short-term outcomes in patients with JE. Vaccination for adults should be initiated.

## 1. Introduction

Japanese encephalitis (JE) is a kind of mosquito-borne viral encephalitis caused by the Japanese encephalitis virus (JEV) (1). JE is one of the most common viral encephalitis in Asia with 70,000 cases and 15,000 deaths every year. Approximately 30–50% of survivors have severe neuropsychiatric sequelae. China accounts for 50% of the reported JE cases (2). Symptomatic patients occur in about 1 in every 250 patients with subclinical infection. The symptoms are mostly non-specific prodromes, such as fever, headache, altered sensorium, and seizures (3). There is no specific treatment other than supportive therapy (4). Vaccination is still the most important method to prevent the disease. According to the early reports, children under the age of 15 were mainly affected in endemic areas (5, 6). The morbidity of JE in adults increases year by year and gradually exceeds that of JE in children due to the vaccination of JE in children. In 2018, 64% of JE cases were adults in China, while 82% of cases were adults in Northern China (7).

It is essential for us to comprehend the differences between adults and children with JE, which may enable us to provide better clinical plans to effectively deal with JE patients. There were few studies that demonstrated the differences in acute complications and short-term outcomes between adults and children in Northern China. To further understand the differences between adults and children, we compared and analyzed the clinical characteristics and short-term outcomes between both groups.

## 2. Patients and methods

### 2.1. Patients

**Figure 1:**
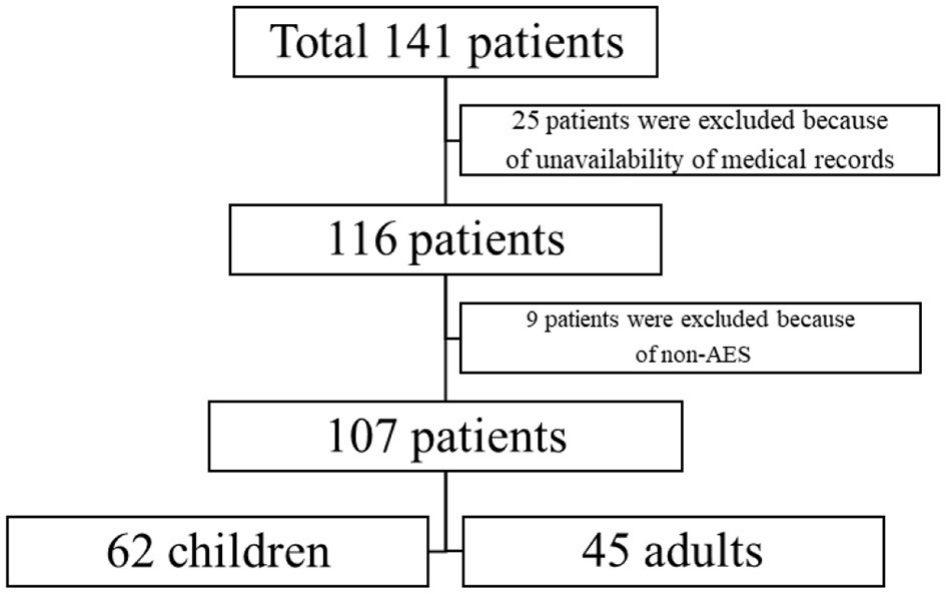
Flowchart of patient inclusion.

The retrospective study consists of 141 diagnosed JE cases of all age groups admitted from August 2006 to October 2019, who were admitted to two medical centers in Northern China, namely Shandong Provincial Hospital and Linyi People's Hospital. Out of the total 141 patients, the data of 107 patients were available (Figure 1). The patients were grouped into the pediatric group (patients < 15 years old) and the adult group (patients ≥ 15 years old).

#### 2.1.1. Inclusion criteria

Patients who satisfied the clinical diagnosis and laboratory criteria.

##### 2.1.1.1. Clinical diagnosis

The clinical diagnosis was based on the presence of clinical criteria of acute encephalitis syndrome (AES) (8, 9), which were as follows: a person of any age, at any time of year, with the acute onset of fever that lasted less than 14 days, and a change in mental status and/or new onset of seizures (excluding simple febrile seizures).

##### 2.1.1.2. Laboratory criteria

The laboratory criteria were as follows: (1) detectable JE-specific IgM in the cerebrospinal fluid (CSF) or serum, (2) evidence of seroconversion or a 4-fold increase of IgM or IgG in the convalescence phase by the ELISA method, (3) isolation of virus from blood, CSF fluid or tissue, or (4) detection of JE-virus genome in serum, plasma, blood, CSF, or tissue.

#### 2.1.2. Exclusion criteria

The exclusion criteria were as follows: (1) Patients who were not in the acute phase (patients who had a history of fever that lasted more than 14 days) (8, 9). (2) Patients with a lack of medical records.

In total, nine patients in the subacute or chronic phase were excluded. Moreover, 25 patients were also excluded due to the unavailability of medical records (Figure 1).

### 2.2. Data collection

A review of medical charts was performed, including details of demographic data, initial clinical features, laboratory data, and imaging findings. In addition, acute complications and treatment were also collected in detail. Short-term outcomes were classified into two types according to the Glasgow Coma Scale (GCS) scores at discharge. Patients with a GCS score >8 at discharge were included in the good outcome (GO) group and those with a GCS score ≤8 or depending on mechanical ventilation were included in the poor outcome (PO) group (10).

### 2.3. Statistical analysis

Baseline patient characteristics were described by the number (percentage) or median (interquartile range [IQR]). The Wilcoxon rank-sum test was used for continuous variables and the Pearson chi-square or Fisher's exact test was used for categorical variables. All statistical analyses were performed using IBM SPSS Statistics for Windows version 25.0 (Armonk, NY). A *P*-value of <0.05 was considered to be statistically significant.

## 3. Results

### 3.1. Demographic and clinical characteristics

The study consisted of 107 patients, with 62 pediatric patients and 45 adult patients. The demographics are shown in Table 1. All patients had at least one positive laboratory test for JE. The median (IQR) age of patients was 10 (5–37) years. The median (IQR) age of pediatric and adult cases was 6 (4–9) years and 43 (24.5–56) years, respectively. Out of a total of 107 patients, 58 were male patients (39 children and 19 adults) and 49 were female patients (23 children and 26 adults), which was similar in both groups. The disease was endemic in summer and autumn from July to October, especially in August and September, which coincided with the mosquito spreading season. Only 34 patients had a record of vaccination against JE, of which 20 children (20/28, 71.4%) and four adults (4/6, 66.7%) were vaccinated. No significant differences between both groups were observed in sex, epidemic season, and vaccination.

**Table 1:** Demographic and clinical characteristics were recorded in both groups of pediatric and adult patients with JE.

**Characteristics (*n =* 107)**	***n* (%) or median (IQR)**	**Pediatric (*n =* 62)**	**Adult (*n =* 45)**	***P*-value**
**Demographic**				
Median age	10 (5–37)	6 (4–9)	43 (24.5–56)	
Male	58 (54.2)	39 (61.3)	19 (44.4)	0.084
Month of onset				0.097
7	3 (2.8)	1 (1.6)	2 (4.4)	
8	33 (30.8)	14 (22.6)	19 (42.2)	
9	59 (55.1)	39 (62.9)	20 (44.4)	
10	12 (11.2)	8 (14.3)	4 (8.9)	
Vaccination^a^	24/34 (70.6)	20/28 (71.4)	4/6 (66.7)	1
**Clinical feature**				
Fever	107	62	45	
Altered consciousness	73 (68.2)	44 (71)	29 (64.4)	0.474
Headache	58 (54.2)	29 (46.8)	29 (64.4)	0.07
Convulsion	43 (40.2)	34 (54.8)	9 (20)	< 0.001^*^
Dyspnea	20 (18.7)	9 (14.5)	11 (24.4)	0.193
Nausea/Vomiting	18 (16.8)	10 (16.1)	8 (17.8)	0.882
Dizziness	6 (5.6)	2 (3.2)	4 (8.9)	0.236
Limb weakness	6 (5.6)	3 (4.8)	3 (6.7)	0.694
Altered sensorium	3 (2.8)	0	3 (6.7)	0.071
**Complications**				
Pulmonary infection	44 (41.1)	19 (30.6)	25 (55.6)	0.01^*^
Respiratory failure	17 (15.9)	9 (14.5)	8 (17.8)	0.649
Upper gastrointestinal bleeding	11 (10.3)	4 (6.5)	7 (15.6)	0.227
Septicemia	10 (9.3)	10 (16.1)	0	0.013^*^
Mycoplasma infection^b^	5 (5/26, 19.2)	5 (5/25, 20)	0 (0/1, 0)	1
Electrolyte disorder	7 (6.5)	3 (4.8)	4 (8.9)	0.451
Myocardial damage	4 (3.7)	4 (6.5)	0	0.137

IQR, interquartile range;

* *p* < 0.05.

a Thirty-four patients had records of vaccination.

b Twenty-six patients had serological records of mycoplasma pneumoniae.

The disease lacks typical clinical manifestations and varies in severity. The clinical characteristics are also shown in Table 1. The most common symptoms were fever (107/107, 100%) and altered consciousness (73/107, 68.2%), followed by headache (58/107, 54.2%). Convulsion (43/107, 40.2%) was more common in 34 children (34/62, 54.8%) than in nine adults (9/45, 20%; *P* ≤ 0.001). Furthermore, neurological symptoms including dizziness (6/107, 5.6%), limb weakness (6/107, 5.6%), and altered sensorium (3/107, 2.8%) were also analyzed. Non-neurological symptoms included dyspnea (20/107, 18.7%) and nausea or vomiting (18/107, 16.8%). There were no significant differences between both groups in neurological and non-neurological symptoms except convulsion. Comorbidities such as hypertension and diabetes mellitus were only observed in adults. Overall, eight adults (8/45, 7.5%) suffered from hypertension, and five adults (5/45, 4.7%) had diabetes mellitus.

The acute complications were also recorded in detail, as shown in Table 1. The most common complications were pulmonary infection (44/107, 41.1%) and respiratory failure (17/107, 15.9%) in both groups. The incidence of pulmonary infection was higher in 25 adults (25/45, 55.6%) than in 19 children (19/62, 30.6%; *P* = 0.01). Septicemia was only observed in 10 children (10/62, 16.1%; *P* = 0.013). There were only 26 patients with serological records of mycoplasma pneumoniae, including 25 children and one adult. The serological results were positive in five children (5/25, 20%). The results were negative in 20 children and one adult. There was no significant difference between children and adults with serological records of mycoplasma pneumoniae [5 (5/25) vs. 0 (0/1), *P* = 1]. Furthermore, upper gastrointestinal bleeding (11/107, 10.3%), electrolyte disorder (7/107, 6.5%), and myocardial damage (4/107, 3.7%) were analyzed in both groups. No significant differences between both groups were observed in complications except pulmonary infection and septicemia.

To further evaluate the influence of acute complications such as pulmonary infection, the patients were divided into two groups according to whether they had a pulmonary infection. The results are shown in Table 2. Dyspnea was more common in 13 patients with pulmonary infection (13/44, 29.5%) than in seven patients without infection (7/63, 11.1%; *P* = 0.016). Respiratory failure was more common in 13 patients with infection (13/44, 29.5%) than in four patients without infection (4/63, 6.3%; *P* = 0.001). Upper gastrointestinal bleeding was more common in 10 patients with infection (10/44, 22.7%) than in one patient without infection (1/63, 1.6%; *P* = 0.001). A total of 26 patients with infection (26/44, 59.1%) required mechanical ventilation, which was more common than in 12 patients without infection (12/63, 19%; *P* < 0.001). The GCS scores at admission in patients with pulmonary infection (7.5, 5.25–10.75) were lower than in patients without pulmonary infection (11, 8–13; *P* < 0.001). At the same time, the GCS scores at discharge in patients with pulmonary infection (7, 4–12.75) were lower than in patients without pulmonary infection (14, 10–14; *P* < 0.001). The poor outcome was more common in 24 patients with infection (24/44, 54.5%) than in 11 patients without infection (11/63, 17.5%; *P* < 0.001). Overall, 16 patients with pulmonary infection (16/44, 36.4%) were admitted to the intensive care unit (ICU) for supportive care, which was more common than in nine patients without patients (9/63, 14.3%; *P* = 0.008).

**Table 2:** Clinical characteristics were recorded in both groups with pulmonary infection and without pulmonary infection.

**Characteristics (*n =* 107)**	***n* (%) or median (IQR)**	**No infection (*n =* 63)**	**Infection (*n =* 44)**	***P*-value**
Dyspnea	20 (18.7)	7 (11.1)	13 (29.5)	0.016^*^
Respiratory failure	17 (15.9)	4 (6.3)	13 (29.5)	0.001^*^
Upper gastrointestinal bleeding	11 (10.3)	1 (1.6)	10 (22.7)	0.001^*^
Mechanical ventilation	38 (35.5)	12 (19)	26 (59.1)	< 0.001^*^
Poor outcome	35 (32.7)	11 (17.5)	24 (54.5)	< 0.001^*^
ICU admission	25 (23.4)	9 (14.3)	16 (36.4)	0.008^*^
GCS at admission	9 (7–13)	11 (8–13)	7.5 (5.25–10.75)	< 0.001^*^
GCS at discharge	12 (6–14)	14 (10–14)	7 (4–12.75)	< 0.001^*^

IQR, interquartile range;

* *P* < 0.05.

### 3.2. Image and laboratory findings

The CSF findings were recorded and are shown in Table 3. CSF samples were obtained from 68 patients. The median (IQR) CSF protein was 0.57 (0.35–0.76) g/L, which was higher in adults than in children (*P* = 0.004). A total of 42 patients (42/68, 61.8%) had CSF protein elevation (CSF protein elevation: >0.45 g/L), which was more common in 22 adults (22/26, 84.6%) than in 20 children (20/42, 47.6%; *P* = 0.002). Nearly half of the patients (35/68, 51%) had CSF pleocytosis. Most patients (58/68, 85.3%) were lymphocyte predominant.

**Table 3:** Summary of laboratory findings of pediatric and adult patients with JE.

**CSF analysis (*n =* 68)**	***n* (%) or median (IQR)**	**Pediatric (*n =* 42)**	**Adult (*n =* 26)**	***P*-value**
CSF protein (g/L)	0.57 (0.35–0.76)	0.43 (0.31–0.73)	0.62 (0.051–0.094)	0.004^*^
^a^CSF protein elevation	42 (61.8)	20 (47.6)	22 (84.6)	0.002^*^
CSF WBC (*10^6^/L)	53 (22.25–138.75)	41 (19–144.75)	84.5 (35–130.75)	0.096
^a^Pleocytosis	35 (51)	19 (45.2)	16 (61.5)	0.191
^a^MMN predominant	58 (85.3)	35 (83.3)	23 (88.5)	0.562
**Blood exams (*****n** =* **78)**	***n*** **(%) or median (IQR)**	**Pediatric (*****n** =* **41)**	**Adult (*****n** =* **37)**	
Platelet (*10^9^/L)	260.5 (176–331.5)	308 (252.5–416.5)	177 (158–253.5)	< 0.001^*^
Albumin (g/L)	39.15 (35.2–41.2)	39.8 (36.05–42.7)	37.6 (34.4–40.8)	0.064
Creatinine (umol/L)	43.7 (32.15–57.26)	35.29 (27.29–44.2)	53 (44.5–68.35)	< 0.001^*^

a CSF protein elevation: >0.45 g/L; pleocytosis, CSF WBC count >8^*^10^6^cells/L.

* *p* < 0.05. CSF, cerebrospinal fluid; WBC, white blood cell; MMN, monomorphonuclear cell.

The blood findings were also recorded, as shown in Table 3. Unlike the common hemogram in patients affected by other vector-borne viral diseases with thrombocytopenia (11), only three patients had thrombocytopenia (platelet < 100^*^10^9^/L). The median (IQR) platelet was 260.5 (176–331.5) ^*^ 10^9^/L, which was higher in children than in adults (*P* < 0.001). The median (IQR) creatinine was 43.7 (32.15–57.26) umol/L, which was higher in adults than in children (*P* < 0.001).

The brain magnetic resonance imaging (MRI) results of 89 patients were available. The image findings are shown in Table 4. According to the results, the thalamic (51/89, 57.3%), basal ganglion (16/89, 18%), and midbrain (14/89, 15.7%) were the common lesion areas in both children and adults. A hippocampal lesion was noted in 10 patients (10/89, 11.2%). A hippocampal lesion was more common in seven adults (7/33, 21.2%) than in three children (3/56, 5.4%), although the difference was not statistically significant. Other lesion areas such as the pons (7/89, 7.9%), medulla (4/89, 4.5%), and occipital lobe (4/89, 4.5%) were also recorded in the study. There was no significant difference between children and adults in the image findings.

**Table 4:** MRI findings of pediatric and adult patients with JE.

**Area (*n =* 89)**	***n* (%)**	**Pediatric (*n =* 56)**	**Adult (*n =* 33)**	***P*-value**
Thalamic	51 (57.3)	33 (58.9)	18 (54.5)	0.686
Basal ganglion	16 (18)	8 (14.3)	8 (24.2)	0.237
Hippocampus	10 (11.2)	3 (5.4)	7 (21.2)	0.052
Midbrain	14 (15.7)	12 (21.4)	2 (6.1)	0.054
Pons	7 (7.9)	5 (8.9)	2 (6.1)	1
Medulla	4 (4.5)	3 (5.4)	1 (3)	1
Frontal lobe	14 (15.7)	12 (21.4)	2 (6.1)	0.054
Temporal lobe	8 (9.0)	6 (10.7)	2 (6.1)	0.721
Parietal lobe	5 (5.6)	2 (3.6)	3 (9.1)	0.355
Occipital lobe	4 (4.5)	4 (7.1)	0	0.292

### 3.3. Clinical course and short-term outcome

The clinical course and short-term outcome were recorded and are shown in Table 5. The median (IQR) time from symptom onset to admission was 4 (3–7) days, which was longer in adults (6, 3.5–8 days) than in children (4, 3–6 days) (*P* = 0.019). The median (IQR) time of hospital duration was 16 (10–23) days, which was longer in children (19, 12.73–23 days) than in adults (12, 7–23 days) (*P* = 0.043). As for the acute complications, 38 patients (38/107, 35.5%) required mechanical ventilation, which was more common in 21 adults (21/45, 46.7%) than in 17 children (17/62, 27.4%; *P* = 0.04). The median (IQR) time of ventilation was 9 (5–13.75) days. In total, 25 patients (25/107, 23.4%) were admitted to ICU primarily due to the rapid progression of the disease, with a median (IQR) ICU stay of 8 (5–15.5) days. The median (IQR) time from symptom onset to ICU was 6 (3.5–8) days, which was longer in adults (7.5, 4.5–9 days) than in children (4, 3–6 days) (*P* = 0.027). Patients received immunotherapy, including glucocorticoid (86/107, 80.4%), intravenous immunoglobulin (52/107, 48.6%), and combination therapy (32/107, 29.9%). Pediatric patients who received immunotherapy were more common (*P* < 0.05).

**Table 5:** Clinical course and outcome of pediatric and adult patients with JE.

**Characteristics (*n =* 107)**	***n* (%) or median (IQR)**	**Pediatric (*n =* 62)**	**Adult (*n =* 45)**	***P*-value**
Days from onset to admission	4 (3–7)	4 (3–6)	6 (3.5–8)	0.019^*^
Hospital duration (days)	16 (10–23)	19 (12.73–23)	12 (7–23)	0.043^*^
Mechanical ventilation	38 (35.5)	17 (27.4)	21 (46.7)	0.04^*^
Days of mechanical ventilation	9 (5–13.75)	8 (5–12)	9 (5.5–17)	0.485
ICU admission	25 (23.4)	13 (21)	12 (26.7)	0.492
ICU duration (days)	8 (5–15.5)	8 (5.5–13.75)	8 (5–23.75)	0.934
Days from onset to ICU	6 (3.5–8)	4 (3–6)	7.5 (4.5–9)	0.027^*^
Days from admission to ICU	0 (0–0.5)	0 (0–0.5)	0 (0–0.75)	0.799
^a^Days from symptoms to immunotherapy	4 (3–7)	4 (3–5.75)	6.5 (3–7.25)	0.149
Glucocorticoids	86 (80.4)	56 (90.3)	30 (66.7)	0.002^*^
Days of glucocorticoids	8.5 (5–14.25)	9 (5–15)	7.5 (3.75–14)	0.598
IVIg	52 (48.6)	47 (75.8)	5 (11.1)	< 0.001^*^
Days of IVIg	3 (2–3)	3 (2–3)	3 (2–11.5)	0.202
Combination therapy	32 (29.9)	28 (45.2)	4 (8.9)	< 0.001^*^
Days of combination therapy	3 (2–3)	3 (2–3)	3 (1.5–10.5)	0.606
GCS at admission	9 (7–13)	9.5 (7–13)	7 (6–13)	0.592
GCS at discharge	12 (6–14)	13 (10.75–14)	7 (3.5–13)	< 0.001^*^
^b^Poor outcome	35 (32.7)	11 (17.7)	24 (53.3)	< 0.001^*^
Death	2 (1.9)	0	2 (4.4)	0.175

a Eighty-six patients received immunotherapy.

b Poor outcome: GCS ≤ 8;

* *p* < 0.05. ICU, intensive care unit; GCS, Glasgow Coma Scale; IVIg, intravenous immune globulin.

Short-term outcomes were evaluated by the GCS score at discharge. The median (IQR) GCS scores at admission were 9 (7–13), which was similar in children (9.5, 7–13) and adults (7, 6–13). The median (IQR) GCS scores at discharge were 12 (6–14), which was lower in adults (7, 3.5–13) than in children (13, 10.75–14; *P* < 0.001). A total of 35 patients (35/107, 32.7%) with poor outcomes (GCS ≤ 8) were observed in both groups, with 24 adults (24/45, 53.3%) and 11 children (11/62, 17.7%; *P* < 0.001).

## 4. Discussion

We conducted the study to analyze the differences between children and adults with JE in clinical features and short-term outcomes. We found that convulsion was more common in children than in adults. Moreover, pulmonary infection was more common in adults than in children. Patients with pulmonary infection were more likely to develop other acute complications such as upper gastrointestinal bleeding and respiratory failure. In addition, patients with pulmonary infection required more mechanical ventilation and ICU support and had worse short-term outcomes. In our study, the proportion of immunotherapy was higher in children. The short-term outcome of adults was worse than that of children.

In this study, no significant difference was seen between both groups with JE in sex and endemic season. The proportion of male patients is higher than that of female patients, which is consistent with previous studies (12). JE cases were distributed mainly between July and October, especially in August and September, which was consistent with mosquito transmission season (7). It is necessary to take protective measures such as using bed nets. The initial clinical symptoms were flu-like symptoms including fever, altered consciousness, and headache. At the initial stage of JE, patients lacked typical clinical manifestations (10). The occurrence of convulsion in children was higher than that in adults (*P* < 0.001). The result was consistent with other studies (13).

In addition to central nervous system (CNS) abnormality, other systems such as the digestive system and the respiratory system were also involved (14). In another study, a significant correlation between abnormal breathing and upper gastrointestinal bleeding was observed (15). Therefore, our study compared the acute complications between children and adults.

According to our study, the most prominent problems during the course of JE were pulmonary infection and other critical secondary complications which needed intensive care support (16). Septicemia was more common in children than in adults (*P* = 0.013). Septicemia is a type of bloodstream infection that causes significant morbidity and mortality. Septicemia was more common in children than in adults throughout the world (17). Moreover, respiratory failure, upper gastrointestinal bleeding, electrolyte disorder, and myocardial damage were also noted. Some complications such as upper gastrointestinal bleeding were related to prognosis (15). It is essential for us to note the complications of patients during hospitalization. To further analyze the influence of complications, the patients were divided into two groups according to whether they had a pulmonary infection. Patients with lower GCS scores at admission were attended to develop pulmonary infections. Dyspnea and respiratory failure were more common in patients with pulmonary infections. At the same time, patients with pulmonary infections needed more supportive care such as mechanical ventilation and ICU admission. The short-term outcome of patients with infection was worse than that of patients without infection (18). According to another study, abnormal breathing patterns predicted a poor outcome, which might explain the phenomenon that patients with pulmonary infection had worse outcomes. Thus, patients with JE require timely treatment of neurological symptoms and acute complications.

According to our study, serological results were noted. The elevated level of CSF protein in pediatric and adult patients in the present study conformed to the published article (19). A previous study found that CSF protein levels were related to neurological symptoms. This might suggest that abnormal intrathecal protein synthesis would lead to poor outcomes (10). The level of CSF leukocyte was higher in adults than in children, although the difference was not significant. The result was also consistent with a previous study. Nearly all patients with JE were lymphocyte predominant, which was accorded with other types of viral encephalitis (14). As for the blood examinations, we compared the levels of platelet, albumin, and creatinine. There were significant differences between both groups in platelet and creatinine. The abnormalities of the serum tests coincided with the previous articles (14).

A comparison of image findings was analyzed in the study. As mentioned in another study, the most common lesion areas were thalamic, basal ganglion, and midbrain in both groups with no difference (20). However, it is worth noting that the proportion of hippocampal damage was higher in adults. A study in Gansu Province found that the proportion of adaptive behavior impairments and intellectual disability in adults with JE was higher than that in children although there was no difference between both groups (21). The imaging results of the study may explain the difference. In addition, another study pointed out that the dynorphin system in the hippocampus was related to seizures. Seizures were more common in adults than in pediatric patients (14, 22). However, the sample size was small, which needed further support study. Lesions of the temporal lobe were also observed in our study. Combined with epileptic symptoms, it is easy to confuse the diagnosis of herpes simplex encephalitis and JE (23), so it is necessary to detect other pathogens.

JE is a severe neuropsychiatric disorder with high morbidity and mortality, but there is no effective treatment. Vaccination against JE is important. According to another research, antiviral therapy combined with immunotherapy can reduce mortality due to severe JE (24). In the study, we compared the clinical course and treatments between both groups. The proportion of immunotherapy in children was higher than that in adults. Moreover, we compared the proportion of ICU admission and median days from symptom onset to ICU admission. The median days from symptom onset to ICU admission in adults were longer than those in children. It seemed that children with JE were more likely to be treated regularly.

Because of the large time span and the retrospective data collection, it was difficult to collect the long-term prognosis of patients after discharge. Some studies pointed out that the GCS score at admission was associated with mortality, and the GCS score at discharge had a certain statistical significance for the long-term prognosis (13, 15). Therefore, we took the GCS score at discharge as the endpoint for the short-term outcome and hoped to predict the long-term prognosis.

In the study, the GCS scores at discharge in adults were lower than those in children, and the proportion of poor outcomes (PO) in adults was higher than that in children. In some studies, researchers tried to analyze the related factors of prognosis (13, 25, 26). As far as our study was concerned, we analyzed the possible reasons. First, mechanical ventilation in adults was more common than in pediatric patients. A study found that mechanical ventilation was associated with mortality. Another study pointed out that the proportion of poor prognosis in JE patients with mechanical ventilation was higher than that in patients without mechanical ventilation (16, 27). Second, the time from onset to hospitalization in adults was longer than that in children, and the duration of treatment in adults was shorter than that in children. In a study, the illness for 7 days or more gave the best prediction of outcome (28). Third, children received more standardized immunotherapy and symptomatic supportive treatments than adults. Fourth, the number of participants in this retrospective study was relatively small.

Although this study confirmed that timely immunotherapy and symptomatic support therapy were effective methods for patients with JE, the cost and sequela were still difficult to improve. Vaccination is still an economical and effective method. According to the latest study of 1,570 cases, the rate of vaccination in children reached 31.3%, while the rate of vaccination in adults was only 0.3% (29). However, there was no significant difference in vaccination between pediatric and adult patients with JE in our study. It might be ascribed to the relatively small sample size. Thus, vaccination for adults should be initiated.

## 5. Conclusion

The short-term outcome of adults was worse than that of children. The short-term outcome of patients with pulmonary infection was worse than that of patients without infection, which means pulmonary infection may be related to poor outcomes. Acute complications such as pulmonary infection should be treated timely in children and adults with JE. Adult patients with JE should be admitted to the hospital early. They also need timely immunotherapy and ICU supportive treatment. Furthermore, vaccination for adults should be initiated.

## Data availability statement

The original contributions presented in the study are included in the article/supplementary material, further inquiries can be directed to the corresponding authors.

## Ethics statement

The studies involving human participants were reviewed and approved by the Ethics Institutional Review Board of Shandong Provincial Hospital. Written informed consent to participate in this study was provided by the participants' legal guardian/next of kin. Written informed consent was obtained from the individual(s), and minor(s)' legal guardian/next of kin, for the publication of any potentially identifiable images or data included in this article.

## Author contributions

Material preparation, data collection, and analysis were performed by FZ, GX, and YL. The first draft of the manuscript was written by FZ. All the authors commented on previous versions of the manuscript. All authors contributed to the conception and design of the study. All the authors read and approved the final manuscript.
